# Ethanol Extract of *Caesalpinia decapetala* Inhibits Influenza Virus Infection In Vitro and In Vivo

**DOI:** 10.3390/v12050557

**Published:** 2020-05-18

**Authors:** Li Zhang, Jungang Chen, Chang Ke, Haiwei Zhang, Shoujun Zhang, Wei Tang, Chunlan Liu, Ge Liu, Si Chen, Ao Hu, Wenyu Sun, Yu Xiao, Minli Liu, Xulin Chen

**Affiliations:** 1 State Key Laboratory of Virology, Wuhan Institute of Virology, Chinese Academy of Sciences, Wuhan 100864, China; zhanglicas000@outlook.com (L.Z.); chenjg@wh.iov.cn (J.C.); xiaobinkechang@hotmail.com (C.K.); hwzhang@wh.iov.cn (H.Z.); tangwei@wh.iov.cn (W.T.); liuchunlan@wh.iov.cn (C.L.); biohazard_1220@hotmail.com (G.L.); chensi880530@outlook.com (S.C.); huaocn@foxmail.com (A.H.); wenyu.sun@hotmail.com (W.S.); xiaoyu.cas@foxmail.com (Y.X.); minli.liu.work@outlook.com (M.L.); 2 University of Chinese Academy of Sciences, Beijing 100049, China; 3 Key Laboratory of Plant Germplasm Enhancement and Specialty Agriculture, Wuhan Botanical Garden, Chinese Academy of Sciences, Wuhan 100864, China; zhangshoujun@wbgcas.cn; 4 Guangdong Provincial Key Laboratory of Virology, Institute of Medical Microbiology, Jinan University, Guangzhou 510630, China

**Keywords:** influenza virus, *caesalpinia decapetala*, antiviral, extract

## Abstract

Influenza virus infections can lead to viral pneumonia and acute respiratory distress syndrome in severe cases, causing significant morbidity and mortality and posing a great threat to human health. Because of the diversity of influenza virus strains and drug resistance to the current direct antiviral agents, there have been no effective drugs as yet to cure all patients infected by influenza viruses. Natural products from plants contain compounds with diverse structures that have the potential to interact with multiple host and virus factors. In this study, we identified the ethanol extract of *Caesalpinia decapetala* (Roth) Alston (EEC) as an inhibitor against the replication of a panel of influenza A and B viruses both on human pulmonary epithelial A549 and human monocytic U937 cells. The animal study revealed that EEC administration reduces the weight loss and improves the survival rate of mice infected with lethal influenza virus. Also, EEC treatment attenuated lung injury and reduced virus titer significantly. In conclusion, we showed that EEC has antiviral activity both in vitro and in vivo, suggesting that the plant *C. decapetala* has the potential to be further developed as a resource of new anti-influenza drugs.

## 1. Introduction

Despite the availability of vaccines and direct-acting antiviral drugs, influenza causes substantial morbidity and mortality every year [1]. Vaccination is limited to the known epidemic strains and takes a long time for preparation [2,3]. There are many problems with the present anti-influenza drugs [4]. Amantadine and amantadine, inhibitors of the M2 ion channel, have been used for decades [5]. With strong side effects and emergence of widespread drug-resistant strains, they are no longer recommended to treat influenza [6]. Current therapy for influenza includes virus neuraminidase inhibitors and polymerase inhibitors [7]. The neuraminidase (NA) inhibitors: oseltamivir, peramivir, and zanamivir, should be administered within 48 h of the onset of symptoms [8]. For advanced and severely ill patients, there is no significant improvement in clinical effect [9]. The most recently approved drug baloxavir targets virus polymerase [10]. However, according to the clinical results, compared with treatment in adults, baloxavir showed only a weak therapeutic effect in the susceptible population that includes children, adolescents, and the elderly, which cannot shorten the course of the disease [11]. Since influenza causes a huge burden on society every year in most countries in the treatment of seasonal and pandemic flu, there is an urgent need to develop new anti-influenza drugs against a broad spectrum of influenza viruses, including the resistant strains [12].

Plants produce a rich and diverse array of natural products; they have had a long history of medicinal use [13,14]. Among them, artemisinin, one of the most famous drugs, was extracted from *Artemisia annua* and has been used to treat malaria [15]. To explore the potential of plant extracts in the treatment of influenza, we collected 600 species of plants from Shen Long Jia, Hubei province, China. By screening the extract library comprising the ethanol extracts of the 600 plants in a U937 cell model against influenza virus infection [16], we found that the ethanol extract of *Caesalpinia decapetala* (Roth) Alston (EEC) has antiviral activity against influenza virus infection. *Caesalpinia decapetala* (Roth) Alston (*C. decapetala*) is a climbing shrub, belonging to the *Caesalpinia* genus of the Fabaceae family, which is distributed all over the world [17]. Chemical investigations revealed that EEC contains a variety of components, such as cassane diterpenoid, spathulenol, lupeol, resveratrol, quercetin, stigmasterol, astragalin, and sitosterol [18,19]. The extract of *C. decapetala* has been reported to have analgesic, anti-oxidant, anti-tumor, and anti-fertility activities [20,21]. The roots of *C. decapetala* are used as a folk medicine to prevent colds, treat bronchitis, and malaria [20]. However, the extract of *C. decapetala* has never been demonstrated experimentally to have antiviral activity. 

In this study, we studied the anti-influenza activity of EEC, both in vitro and in vivo. EEC showed a broad-spectrum inhibitory effect on the replication of all strains of influenza viruses tested on Madin–Darby Canine Kidney (MDCK), A549, and U937 cells. The animal experiments showed that EEC could improve the survival rate of mice infected with lethal influenza virus and decrease the virus titers and pathological damage to the lungs. Our results suggested that EEC has the potential to be a plant-derived drug with further research and development.

## 2. Materials and Methods 

### 2.1. Cell Lines, Virus Strains 

The Madin–Darby Canine Kidney (MDCK) cells (ATCC CCL-34), human pulmonary epithelial (A549) cells (ATCC CCL-185), and human monocyte cell line U973 (ATCC CRL-1593.2) were all preserved in the laboratory. MDCK was cultured in Dulbecco’s modified Eagle’s medium, while A549 and U937 cells were cultured in RPMI-1640 medium, both were supplemented with 10% fetal bovine serum (FBS; Gibco, NY, USA), 100 U/ mL penicillin and 100 U/ mL streptomycin. All these cells were maintained at 37 °C in a 5% CO_2_ incubator. 

Influenza virus strains A/Puerto Rico/8/1934 (H1N1), A/Puerto Rico/8/1934 (H1N1, H274Y oseltamivir-resistant), A/human/Hubei/1/2009 (H1N1), A/human/Hubei/3/2005/(H3N2), A/duck/Hubei/216/1983 (H7N8) and B/human/Hubei/1/2007 (IBV) were provided by the virus collection at Wuhan Institute of Virology, Chinese Academy of Sciences, China and amplified from 10-day-old chicken embryos. The virus titers of different influenza strains were determined using 50% tissue culture infective dose (TCID_50_) assay in MDCK cells.

### 2.2. Preparation of Ethanol Extracts of Plants

The 600 plants were collected from Shen Long Jia, Hubei province, China, followed by extraction with 75% aqueous ethanol. In the confirmation and efficacy study, *C. decapetala* was authenticated and collected from the Wuhan Institute of Botany, Chinese Academy of Sciences. Dried leaves and branches of *C. decapetala* were extracted with 75% aqueous ethanol at room temperature overnight. After filtration, the ethanol extract of *C. decapetala* was stored at 4 °C for further use. The concentration of the extract was determined by the weight of vacuum freeze-dried extract over its original volume.

### 2.3. Cytotoxicity Assay

Cells in 96 well cell culture plates were treated with drugs and cultured at 37 °C for 48 h. The cell viabilities were determined using CellTiter-Glo (Promega, Madison, WI, USA) reagent according to the manufacturer’s protocol. The luminescence intensity was determined using a multi-label plate reader (Wallac Envision, PerkinElmer, MA, USA). Three independent experiments were performed in duplicate for the calculation of 50% cell cytotoxic concentration (CC_50_) using Prism v.6 software.

### 2.4. Antiviral Assay

For the antiviral assay, cells were plated and infected with the influenza virus in the presence or absence of the drug. After incubation at 37 °C for 48 h, the inhibition of viral replication was measured by the modified neuraminidase activity (NA) assay (Ivachtchenko et al., 2013). The fluorescence intensity was measured with a multi-label plate reader (Wallac Envision, PerkinElmer, MA, USA) and was expressed as the 50% effective (inhibitory) concentration (EC_50_). For detection of the production of infectious virus virions, the supernatants, harvested 48 h post-infection (hpi), were titrated through the TCID_50_ assay, and the virus titers were calculated according to the method of Spearman–Karber [22].

### 2.5. Neuraminidase Assay

The fluorescent substrate, 2′-(4-methylumbelliferyl)-a-D-N-acetylneuraminic acid (MUNANA, Sigma, M8639), for the neuraminidase (NA) of influenza viruses was used to detect the levels of NA. Briefly, the virus-containing culture supernatant was transferred to a black opaque 96 or 384 well plate (PerkinElmer, 6005270 or 6007270) and mixed with 20 µmol/L of MUNANA dissolved in MES solution (33 mmol/L 2-[N-morpholino] ethanesulfonic acid and 4 mmol/L CaCl2, pH = 6.5), followed by incubation at 37 °C for 1 h. The reaction was terminated by the addition of stop solution (0.14 mol/L NaOH in 83% ethanol). Fluorescence intensity was measured at an excitation wavelength of 355 nm and an emission wavelength of 485 nm using multi-label plate readers (Envision2103, PerkinElmer, Waltham, MA, USA).

### 2.6. Animal Experiment

BALB/c mice aged 6–8 weeks were purchased from Beijing Vital River Laboratory Animal Technology and raised in the Animal bio-safety level II (ABSL-2) Laboratory of Wuhan Institute of Virology, CAS. All animal experiments were conducted according to the protocol approved by the Animal Care and Use Committee of Wuhan Institute of Virology of the Chinese Academy of Sciences (WIVA08201601). Mice were anesthetized with pentobarbital sodium and then infected with the influenza virus through the nasal cavity. Infected mice were treated by intragastric managed drug once a day for five consecutive days, starting 3 h after infection. During the experimental period of 18 days, the weight changes and survival rates were recorded. On days 3 and 7 post-infection, the lungs of mice were inflation fixed in 10% formalin and paraffin-embedded and then stained with hematoxylin and eosin (H&E). The lung lavage fluids (BALFs) were collected with precooled 0.1% BSA in PBS and followed by centrifugation at 1500 rpm. The virus titers were measured by the TCID_50_ method.

### 2.7. Statistical Analyses

The 50% effective concentrations (EC50s) and 50% cytotoxic concentrations (CC50s), and selective indices (SIs) in the in vitro study were calculated by non-linear regression using GraphPad Prism 6.0. Data were presented as mean ±SD for each point. Differences of averages between control and tests samples in the animal experiments were analyzed using Student’s t test. *p*-Values less than 0.05 were considered statistically significant. 

## 3. Results

### 3.1. EEC Inhibits Influenza Virus Replication on A549 Cells

**Figure 1:**
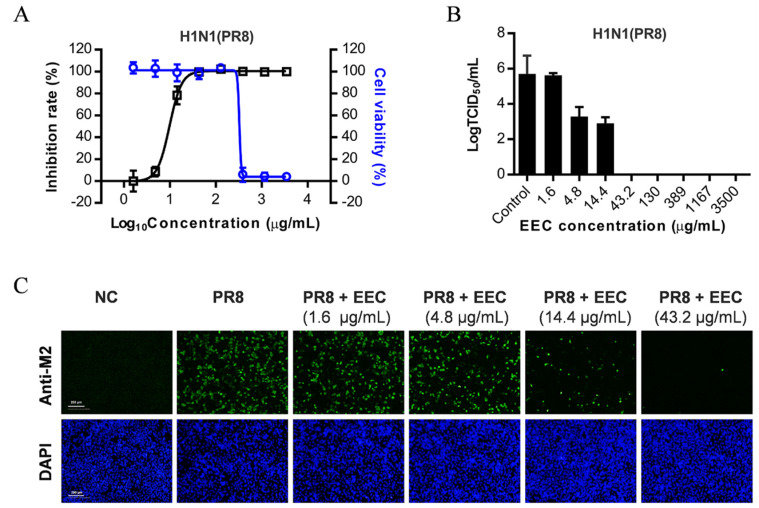
*Caesalpinia decapetala* (Roth) Alston (EEC) inhibits influenza virus replication in A549 cells. A549 cells were infected with influenza virus A/Puerto Rico/8/1934 (H1N1) at an multiplicity of infection (MOI) of 0.25 in the presence or absence of serially diluted EEC. Cells were then incubated at 37 °C for 48 h. Cell viabilities were detected by cytotoxicity assay using Cell Titer-Glo reagent (**A**). The inhibitory effects of EEC on virus replication were determined based on the reduction on NA levels using NA activity assay (A), the production of infectious virions determined by TCID_50_ Assay (**B**), and the expression of M2 determined by IFA using the antibody against virus matrix protein 2 (M2) (**C**).

To screen for natural products of plants with anti-influenza activity, we made ethanol extracts of about 600 plant species from Shen Nong Jia, Hubei province, China. By screening these plant extracts, we found that the ethanol extract of *C. decapetala* (EEC) inhibits the replication of the influenza virus on A549 cells. As shown in Figure 1A, EEC inhibited the H1N1 influenza virus PR8 strain infection on A549 cells, with a CC_50_ and an EC_50_ of 326.4 μg/mL and 9.8 μg/mL, respectively. The inhibition of virion production was also tested, as can be seen in Figure 1B; the results showed that EEC inhibited the production of infectious virions potently and concentration-dependently. At concentrations higher than 43 μg/mL, the production of infectious influenza virions was below the detection limit (10 TCID_50_/mL). The EC_50_ of EEC is about 14 μg/mL. To exclude the possible cytotoxic effect of EEC on virus replication, we used the indirect immunofluorescence assay (IFA) method to measure the cell viability by DAPI staining and to confirm the inhibitory effect by detection of the expression of M2 protein. As shown in Figure 1C, EEC at 43.2 μg/mL and 14.4 μ g/mL inhibited the virus replication completely and about 50%, respectively, which is consistent with that measured by virus production. Importantly, the DAPI staining showed that EEC at these concentrations is not cytotoxic to A549 cells. In conclusion, we demonstrated that EEC inhibits influenza virus replication on A549 cells.

### 3.2. EEC Has a Broad Spectrum of Antiviral Activity against a Panel of Influenza Viruses in A549, U937, and MDCK Cells

**Figure 2:**
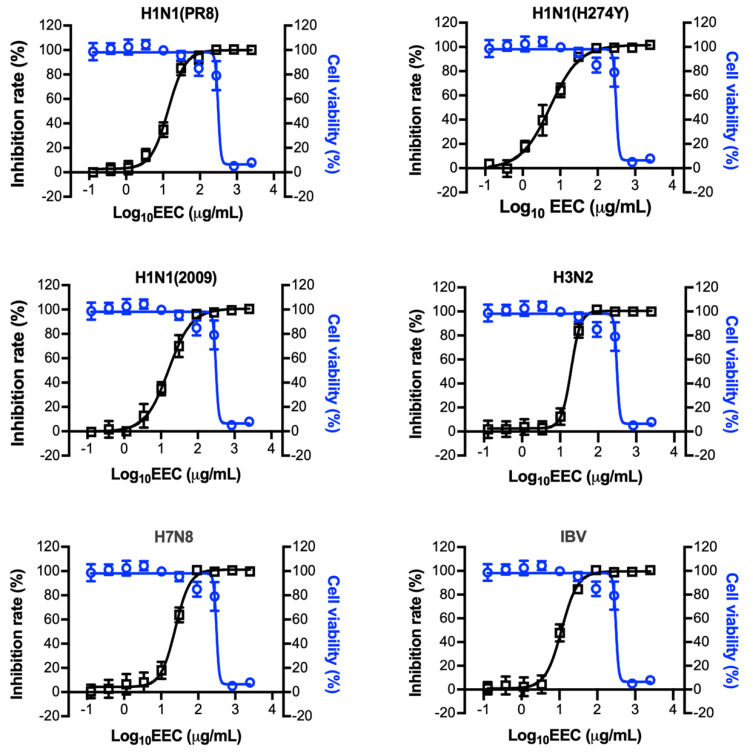
EEC has a broad spectrum of antiviral activities against influenza viruses in A549 cells. A549 cells were infected with influenza viruses A/Puerto Rico/8/1934 (H1N1), A/Puerto Rico/8/1934 (H1N1, H274Y oseltamivir-resistant), A/human/Hubei/1/2009 (H1N1), A/human/Hubei/3/2005 (H3N2), A/duck/Hubei/216/1983 (H7N8) and B/human/Hubei/1/2007 (IBV) at an MOI of 0.25. Serially diluted EEC was added at the same time during the virus infection. The cells were then incubated at 37 °C for 48 h. Cell viabilities and inhibition rates were determined by Cell Titer-Glo and NA activity assay, respectively. The dose-response curves of EEC against different influenza viruses were made using Prism software (GraphPad Software, San Diego, CA, USA).

Human influenza A and B viruses cause annual influenza epidemics, whereas influenza A viruses can also cause sporadic infections or spread worldwide in a pandemic when novel strains emerge in the human population from an animal host [23]. To test the antiviral spectrum of EEC against different types and subtypes of influenza viruses, we infected A549 cells with 0.25 MOI of influenza A/Puerto Rico/8/1934, A/Puerto Rico/8/1934 (H274Y), A/human/Hubei/1/2009 (H1N1), A/human/Hubei/3/2005 (H3N2), A/duck/Hubei/216/1983 (H7N8) and B/human/Hubei/1/2007, respectively. The infected cells by each virus were treated with serially diluted EEC. We found that EEC could inhibit all the influenza viruses, including the oseltamivir-resistant H1N1 (H274Y) virus, on A549 cells at a similar efficiency (Figure 2). During infection of humans, the influenza virus can infect multiple cell types, including epithelial cells and monocytes that are important for the pathogenesis of influenza [24]. We next checked the antiviral effect of EEC on epithelial MDCK cells and monocytic U937 cells against both influenza virus A and B viruses. Surprisingly, EEC was found to inhibit all the influenza virus strains both in MDCK cells and U937 cells. Comparing with canine MDCK cells, EEC inhibits more efficiently the replication of influenza viruses in human A549 and U937 cells (Table 1). 

### 3.3. Determination of the Stage Affected by EEC in the Influenza Virus Life Cycle 

**Figure 3:**
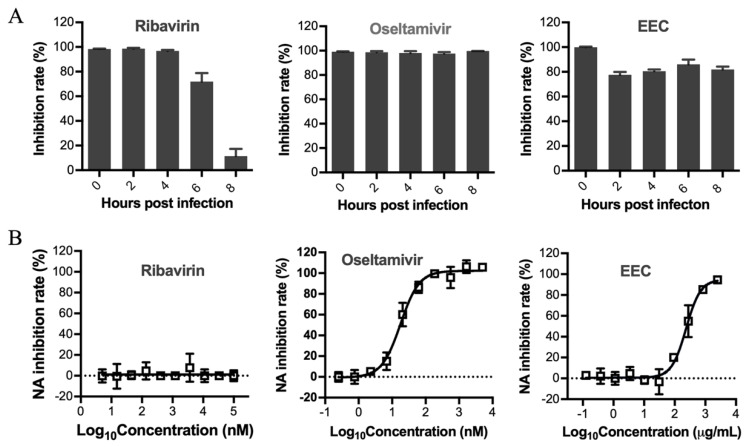
EEC affects the late stage of the influenza virus life cycle by inhibiting neuraminidase activity. The stages of the influenza virus life cycle affected by EEC were determined by time of addition assay and neuraminidase inhibition assay, respectively. (**A**) In the time of addition assay, pre-cooled MDCK cells were infected with PR8 virus at an MOI of 0.1 at 4 °C for 1 h. Three IC_50_ of ribavirin, oseltamivir, and EEC were added in each group at different time points during virus replication on MDCK cells. The supernatants were collected at 12 hpi, and the viral yield was determined by the NA activity assay. (**B**) In the neuraminidase inhibition assay, 95 μL of H1N1 PR8 virus solution of 10^5^TCID_50_/mL was mixed with 5 μL of serially diluted EEC, ribavirin or oseltamivir, respectively. Each reaction was conducted at 37 °C for 1 h in the presence of 20 μM MUNANA, the fluorescence intensity was determined using the EnSpire multi-label plate reader (PerkinElmer, MA, USA).

To determine the stage of the influenza virus life cycle inhibited by EEC, a time-of-addition assay was conducted. MDCK cells were infected with 0.1 MOI of influenza virus at 4 °C for 1 h, followed by cell culture at 37 °C for 12 h. During the cell culture, three EC_50_ of ribavirin, oseltamivir, and EEC were added respectively at different time points and maintained for 12 h. The supernatants were collected, and the virus yields were determined by the NA activity assay. As shown in Figure 3A, similar to oseltamivir, EEC inhibited the virus replication completely when added at all time points until 8 hpi, indicating that EEC may inhibit the very late stage of the influenza virus life cycle, which includes mainly the release of virions that is related to the function of virus neuraminidase. To determine whether EEC inhibits the virus neuraminidase activity, an in vitro neuraminidase inhibition assay was conducted. As shown in Figure 3B, similar to oseltamivir, EEC inhibited the neuraminidase activity concentration-dependently. Taken together, our results show that EEC inhibits the late stage of the influenza virus life cycle. Since the virus neuraminidase activity is required for the release of virions, we speculate that EEC may inhibit the release of influenza viruses.

### 3.4. EEC Protects Mice from Lethal Influenza Virus Infection

**Figure 4:**
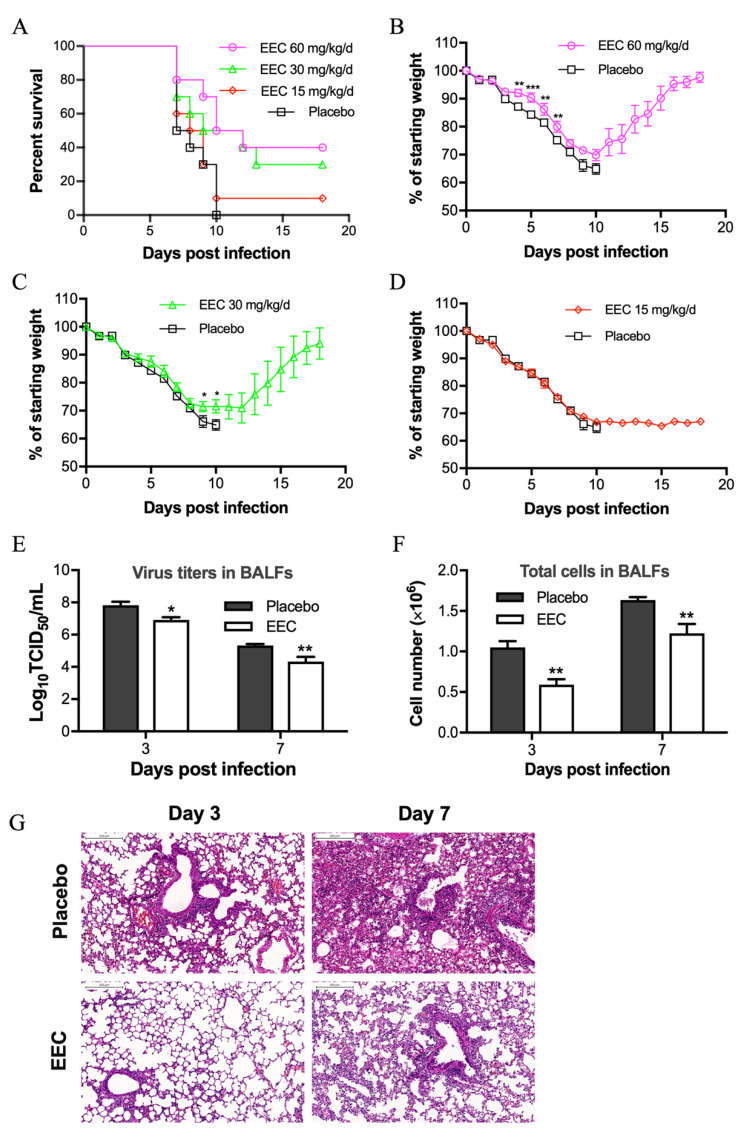
EEC protects mice from lethal influenza virus infection. Six-week-old female BALB/c mice weighing 16–18 g were infected with 4 LD_50_ of H1N1 PR8 virus intranasally. Three hours after infection, four groups of mice (10 mice in each group) were treated intraperitoneally with 60, 30, 15 mg/kg/d of EEC or placebo solution respectively for five consecutive days. Survival rate (**A**) and body weight loss (**B**–**D**) were monitored daily until day 18 post-infection. Lung lavage fluids (BALFs) from the infected mice treated with 60 mg/kg/d of EEC were collected on days 3 and 7 post-infection to monitor virus titers (**E**), and total cell counts (**F**) (mean ±SEM). The data are representative of three independent experiments. (*n* = 3 per group at each time point). * *p* < 0.05; ** *p* < 0.01; *** *p* < 0.001. Sections from lungs of PR8-infected BALB/c mice treated with 60 mg/kg/d of EEC or placebo solution at day 3 and 7 post-infection were H&E-stained (scale bar 200 μm) (**G**).

To further study the therapeutic effect of EEC in vivo, BALB/c mice weighing about 18 g were infected with four LD_50_ of H1N1 PR8 virus by the method of intranasal. After 3 h of infection, each group of mice (*n* = 10) was treated with 60, 30, and 15 mg/kg/d of EEC or placebo solution. The mice of the placebo group began to die on day 7, and all died on day 10 after infection. Compared with the placebo group, the mice treated with 60 mg/kg/d of EEC lost weight relatively slow and began to gain weight significantly after day 10, with a survival rate of 40% (Figure 4A,B). While the mice treated with 30 and 10 mg/kg/d of EEC had 30% and 10% of survival rate, respectively (Figure 4A,C,D). Whereas treatment with the reference drug oseltamivir at 20 mg/kg/d protected 80% of mice from death caused by the same lethal dose of PR8 virus infection. These observations suggest that EEC protects mice from lethal influenza virus infection.

### 3.5. EEC Reduces Virus Titer and Pathogenic Damage in the Lung Mediated by Influenza Virus Infection

Even though the selective indexes of EEC in the inhibitory effects against influenza virus A and B strains in human A549 and U937 cells range from 5 to 52 (Table 1), EEC does protect mice from lethal influenza virus infection. To confirm whether EEC inhibits virus replication in vivo, we next checked the virus titers in the BALFs of the infected mice treated with EEC. As shown in Figure 4E, at day 3 and 7 post-infection, the virus titers in the BALFs of mice treated with 60 mg/kg/d of EEC decreased significantly (about 10 times) compared with that of the placebo group. 

When mice are infected with the influenza virus, a large number of inflammatory cells are often infiltrated in the lung tissue, accompanied by enhanced inflammatory injuries in the lung [25,26]. To examine the total cell infiltration and pathological changes in the lungs of mice infected by lethal influenza and treated or untreated with EEC, the total cell counting in the BALFs and H&E staining of the lung tissue of mice were conducted. As shown in Figure 4F, the total number of cells at day 3 and 7 in BALFs of mice treated with 60 mg/kg/d of EEC decreased by 40% and 25%, respectively. A further pathological examination by H&E staining revealed that the pathogenic damage in the lungs treated with EEC improved significantly at day 3 and 7 post-infection (Figure 4G). We speculate that the reduction in infiltrated cells and pathological damage in the lung may be a result of the decrease in virus titer mediated by the antiviral effect of EEC.

## 4. Discussion/Conclusions

Seasonal influenza is an acute respiratory infection caused by influenza viruses that circulate the world [27]. Influenza A and B viruses cause seasonal epidemics of disease. Currently circulating in humans are subtype A(H1N1) and A(H3N2) influenza viruses [28]. Influenza viruses circulating in animals, e.g., birds, pose threats to human health in that they can develop the ability to infect humans and cause illness [29]. In patients with influenza pneumonia, multiple cell types, including epithelial cells, alveolar cells, and monocytes, are infected by the influenza virus and contribute to direct cytopathogenic and inflammatory damage to the lung [30,31]. In our in vitro study, in addition to the commonly used MDCK cells, we tested the antiviral effect of EEC against a panel of influenza viruses on the epithelial A549 cells and monocytic U937 cells. However, the efficacy of EEC is not as good as oseltamivir (data not shown), but comparable to that of ribavirin (Figure S1).

In addition to human influenza A viruses H1N1 and H3N2, human influenza B virus strains, the 2009 (H1N1) pandemic strain, oseltamivir-resistant strain H1N1 (H274Y), and a bird flu H7N8 were also tested. We did not measure the infectivity using TCID_50_ assay. Instead, we tested the NA activity on the supernatants of the cell culture since we found that the TCID_50_s are correlated pretty well with the NA activities from the same virus-containing supernatants using a fluorescent substrate (Figure S2). Our results showed that EEC has a broad-spectrum of anti-influenza activity in multiple cell types, suggesting EEC has the potential to treat influenza.

Next, the therapeutic effect of EEC was tested on a mouse influenza model. Our results demonstrated that EEC protected mice from lethal influenza infection by increasing the survival rate that is associated with decreased virus titer, cell infiltration, and inflammatory damage to the lung. We assume that the antiviral activity of EEC contributes mainly to the therapeutic effect. Furthermore, a preliminary mechanism study revealed that EEC inhibits the neuraminidase activity and interferes with the late stage of the virus life cycle. It is worth noting that when extracted with pure H_2_O or ethanol, the extract of *C. decapetala* exhibits different antiviral activity and cytotoxicity from that extracted with 75% aqueous ethanol (data not shown). This observation suggests that the extraction methods can influence the efficacy of EEC on its antiviral activity and cytotoxicity. Further optimization of the extraction method for anti-influenza drug development is needed.

Herb medicine has a long history of being used to treat the flu. Many studies have identified bioactive components or compounds in plant extracts that may be useful for treating influenza. A variety of polyphenols, flavonoids, alkaloids, essential oils, and aromatic compounds isolated from medicinal plants and plant extracts have been extensively studied and tested for anti-influenza activity [32]. However, most of the efficacy studies were conducted in vitro; only a few extracts or compounds were demonstrated to be effective in vivo. Among the in vivo studies, Bing et al. reported that the total alkaloid extract from *Commelina communis* showed antiviral activity against influenza virus H1N1 in vivo [33]. Utsunomiya et al. found that glycyrrhizin, an active component of licorice roots, could protect mice exposed to a lethal H2N2 virus through the stimulation of IFN-gamma production by T cells. Glycyrrhizin, by itself, does not inhibit influenza virus replication [34]. Compared with these reported herb medicines, EEC showed similar efficacy to the total alkaloid extract from *Commelina communis*. However, it is less potent than glycyrrhizin in increasing survival rate, though they both can lower the virus titer in the lung of mice for about one log value. When compared to the clinical drug oseltamivir, EEC showed less potency both in increasing survival rate and in lowering virus titer.

EEC, at its current formula, may be not ideal as an anti-influenza drug. However, the plant *C. decapetala* (Roth) Alston can serve as a resource of natural products to be developed as a plant-derived drug against influenza. Further research is in progress to isolate and elucidate the bioactive components or compounds responsible for the antiviral activity of this plant and to determine their mechanisms of action. To the best of our knowledge, this is the first report of the antiviral activity of *C. decapetala* (Roth) Alston. Our findings suggest that the ethnobotanical use of plant drugs may provide benefits in the treatment of influenza, warranting further investigation.

## Figures and Tables

**Table 1:** Inhibition of 6 different influenza virus strains in A549, Madin–Darby Canine Kidney (MDCK), and U937 cells by *Caesalpinia decapetala* (Roth) Alston (EEC).

Virus		H1N1(PR8) ^a^		H1N1(H274Y) ^b^		H1N1(2009) ^c^		H3N2 ^d^		H7N8 ^e^		IBV ^f^	
Cells	CC_50_	EC_50_	SI	EC_50_	SI	EC_50_	SI	EC_50_	SI	EC_50_	SI	EC_50_	SI
A549	311.4 ± 1.1	14.1 ± 1.1	22.1	5.7 ± 1.1	55.1	16.1 ± 1.1	19.4	18.9 ± 1.1	16.5	23.0 ± 1.1	13.6	11.4 ± 1.1	27.2
MDCK	152.7 ± 1.2	17.7 ± 1.1	8.6	6.7 ± 1.2	22.7	7.2 ± 1.1	21.2	24.6 ± 1.2	6.2	24.3 ± 1.1	6.3	80.2 ± 1.1	1.9
U937	719.9 ± 1.1	20.5 ± 1.2	35.1	18.7 ± 1.1	38.5	29.2 ± 1.2	24.7	34.5 ± 1.1	20.9	42.0 ± 1.1	17.1	133.5 ± 1.2	5.4

CC_50_: 50% cytotoxic concentration (μg/mL); EC_50_: 50% inhibition concentration (μg/mL); SI (Selective Index): the ratio of CC_50_/IC_50_. ^a^ A/PuertoRico/8/1934 (H1N1); ^b^ A/PuertoRico/8/1934 (H1N1, H274Y, oseltamivir resistant); ^c^ A/Human/Hubei/1/2009(H1N1); ^d^ A/human/Hubei/3/2005(H3N2);.^e^ A/Duck/Hubei/216/1983(H7N8); ^f^ B/human/Hubei/1/2007.

## Supplementary Materials

Supplementary materials can be found at https://www.mdpi.com/1999-4915/12/5/557/s1. Figure S1: Antiviral effects of ribavirin against influenza virus PR 8 were determined in A 549 U 937 and MDCK cells; Figure S2: Correlation analysis of the TCID 50 and neuraminidase (NA) activity of influenza virus solutions produced in cell culture.
