# Supplementary material for: Ethanol Extract of Caesalpinia decapetala Inhibits Influenza Virus Infection In Vitro and In Vivo
Source: Viruses. 2020 May 18;12(5):557. doi: 10.3390/v12050557 (PMC7290740; doi:10.3390/v12050557)
Supplement: Supplementary file 1 [file viruses-12-00557-s001.pdf]

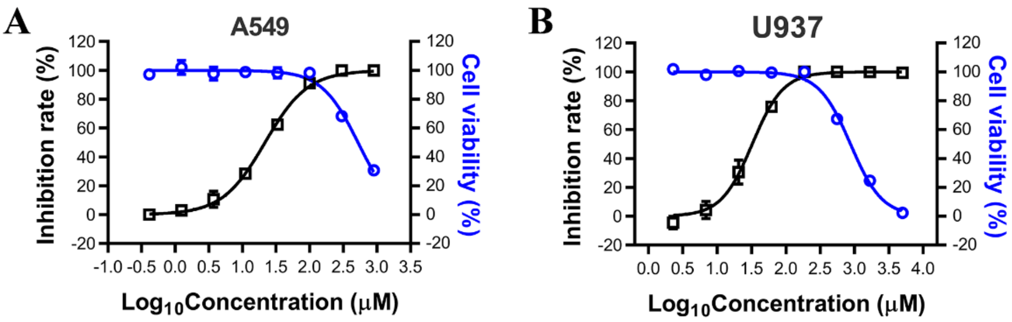

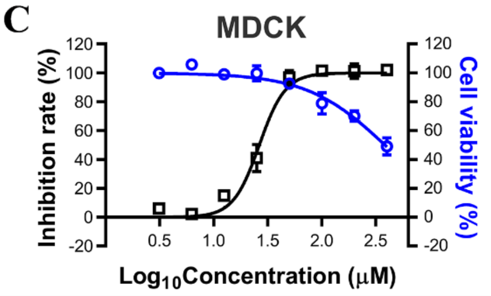

D Table: Anti-influenza effects of ribavirin in different cells *in vitro*.

	CC_{50}	EC_{50}	SI
A549	523.8	21.6	24.2
MDCK	384.1	26.3	15.1
U937	871.2	32.3	26.9

Supplemental Figure 1. Antiviral effects of ribavirin against influenza virus PR8 were determined in A549, U937, and MDCK cells. Ribavirin was used as a reference drug and tested for its antiviral activity and cytotoxicity as described in the Methods section on A549 (A), U937 (B), and MDCK (C) cells. The EC50s and CC50s were determined and summarized in D.

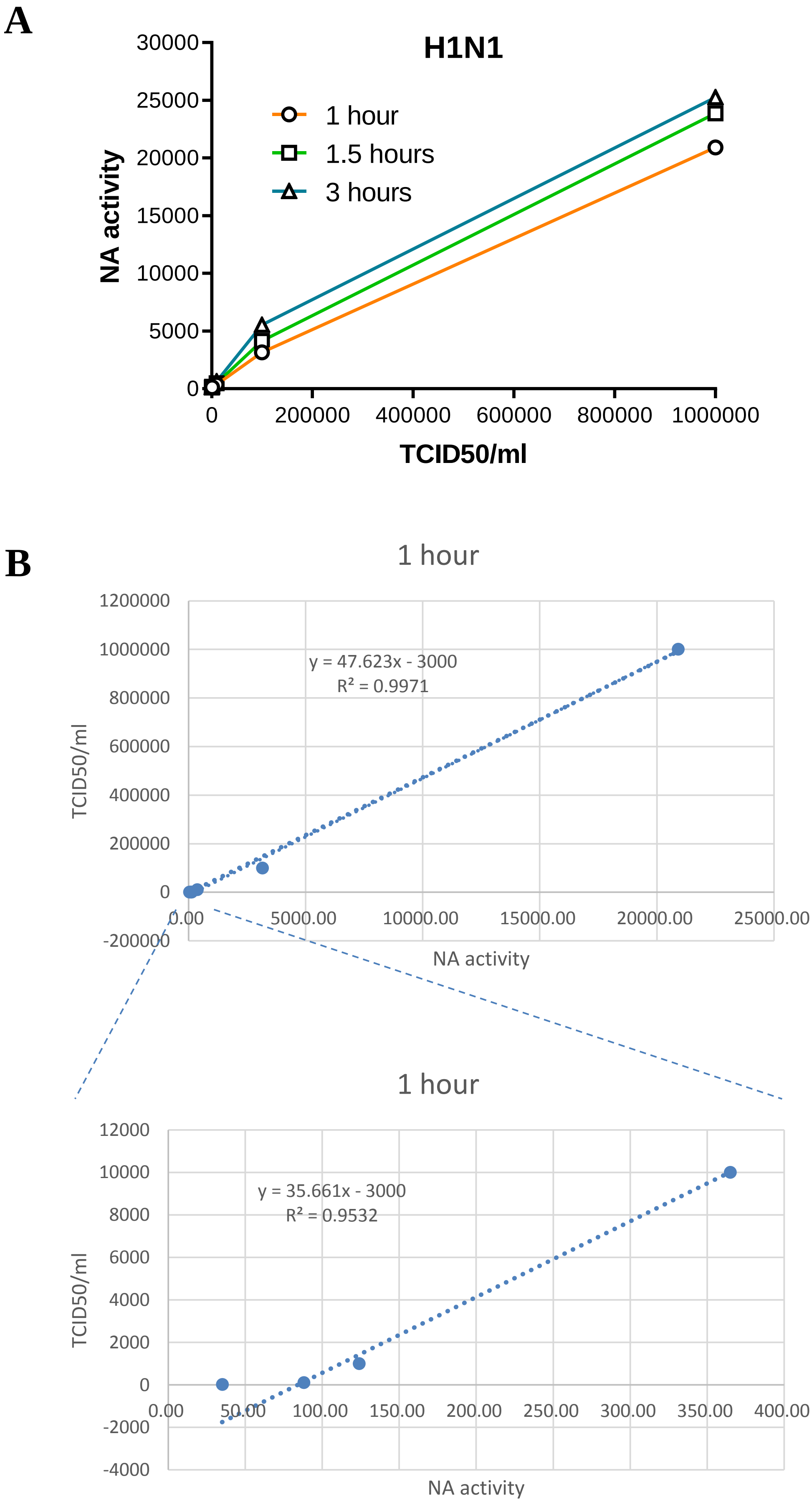
A
H1N1
NA activity
30000
25000
20000
15000
10000
5000
0
1 hour
1.5 hours
3 hours
0
200000
400000
600000
800000
1000000
TCID50/ml
B
1 hour
1200000
1000000
800000
600000
400000
200000
0
-200000
TCID50/ml
y = 47.623x - 3000
R² = 0.9971
0.00
5000.00
10000.00
15000.00
20000.00
25000.00
NA activity
1 hour
12000
10000
8000
6000
4000
2000
0
-2000
-4000
TCID50/ml
y = 35.661x - 3000
R² = 0.9532
0.00
50.00
100.00
150.00
200.00
250.00
300.00
350.00
400.00
NA activity

C

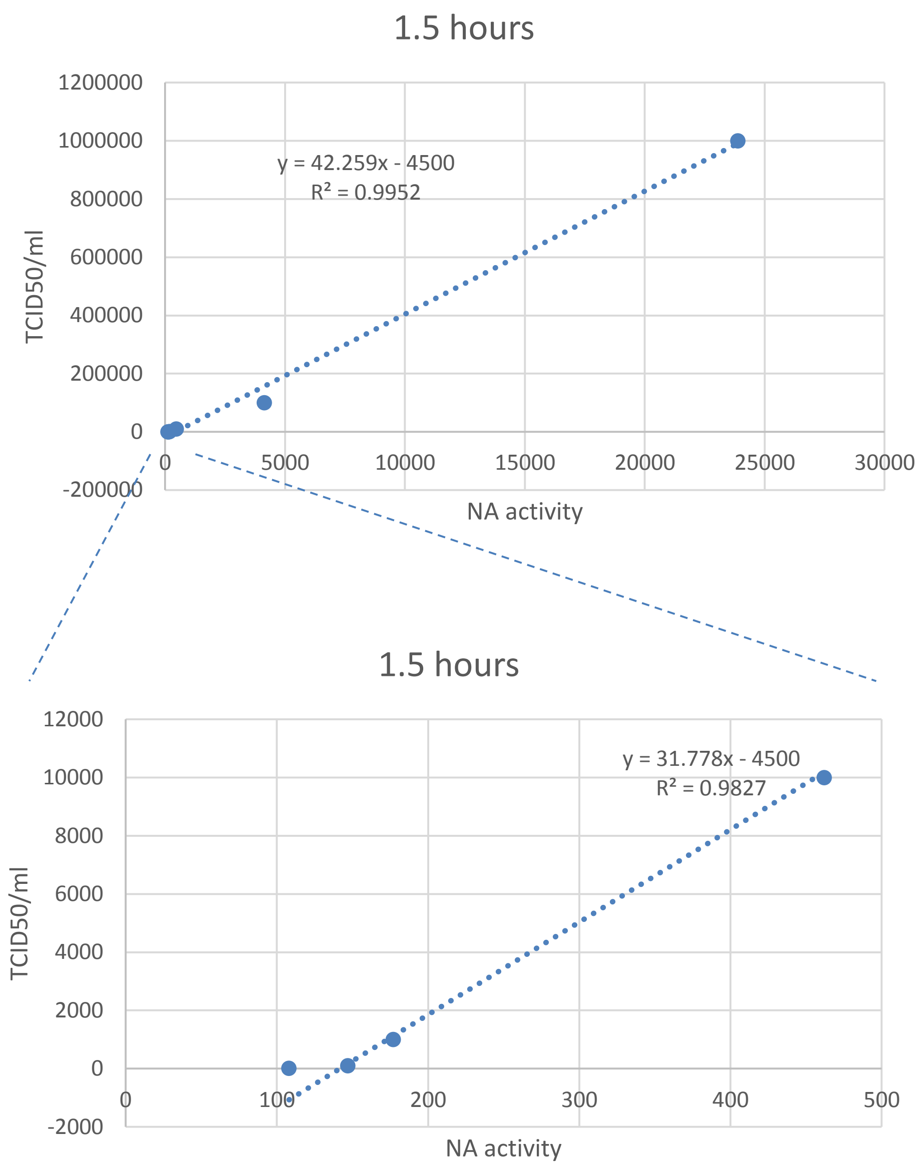

1.5 hours
1200000
1000000
800000
600000
400000
200000
0
-200000
TCID50/ml
0
5000
10000
15000
20000
25000
30000
NA activity
y = 42.259x - 4500
R² = 0.9952
1.5 hours
12000
10000
8000
6000
4000
2000
0
-2000
TCID50/ml
0
100
200
300
400
500
NA activity
y = 31.778x - 4500
R² = 0.9827

D

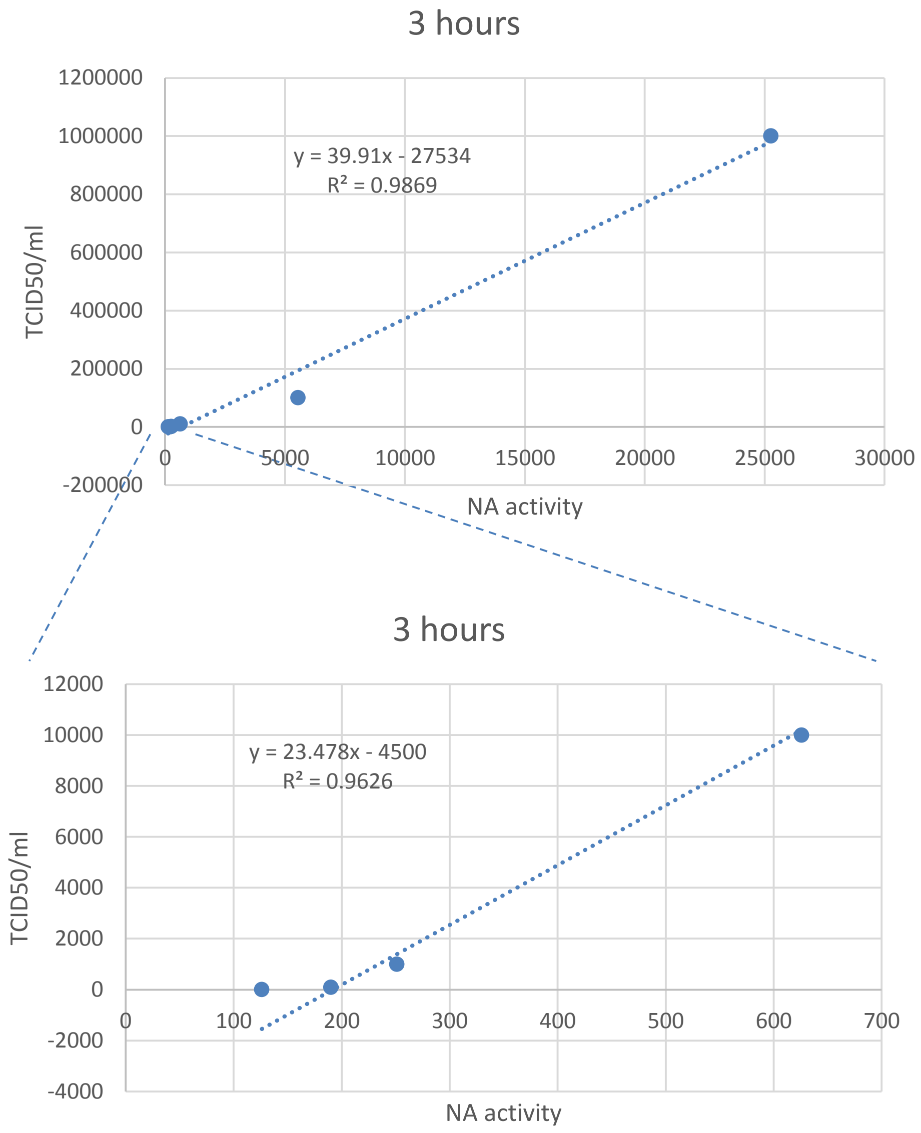

Supplemental Figure 2. Correlation analysis of the TCID50 and neuraminidase (NA) activity of influenza virus solutions produced in cell culture. (A) TCID50s are correlated with NA activity. Influenza PR8 virus stock solution was serially diluted to prepare virus solutions of 10, 100, 1000, 10000, 100000, and 1000000 TCID50/ml. Forty µl of each virus solution at different titer was mixed with 20 µl of the fluorescent substrate, 2'-(4-Methylumbelliferyl)-a-D-N-acetylneuraminic acid (MUNANA, Sigma, M8639), on a black opaque 96 (PerkinElmer, 6005270). The reactions were incubated at 37 ℃ for 1 to 3 hours. The fluorescence intensity was measured at an excitation wavelength of 355 nm and an emission wavelength of 485 nm using multi-label plate readers (Envision2103, PerkinElmer, MA, USA). To further analyse the correlation of TCID50 and NA of influenza virus solutions over a wide range of virus titers and NA activities, the data determined using the NA assay for 1 hour (B), 1.5 hours (C), and 3 hours (D) were shown individually. An equation for each data sets was obtained by linear regression analysis with a coefficient of correlation of > 0.95 using Microsoft Excel 2016.
